# Transcriptomic profiling to identify genes involved in *Fusarium* mycotoxin Deoxynivalenol and Zearalenone tolerance in the mycoparasitic fungus *Clonostachys rosea*

**DOI:** 10.1186/1471-2164-15-55

**Published:** 2014-01-22

**Authors:** Chatchai Kosawang, Magnus Karlsson, Dan Funck Jensen, Adiphol Dilokpimol, David B Collinge

**Affiliations:** 1 Department of Plant and Environmental Sciences, University of Copenhagen, Copenhagen, Denmark; 2 Uppsala BioCenter, Department of Forest Mycology and Plant Pathology, Swedish University of Agricultural Sciences, Uppsala, Sweden

**Keywords:** *Clonostachys rosea*, cDNA library, Fusarium mycotoxins, Deoxynivalenol, Zearalenone

## Abstract

**Background:**

*Clonostachys rosea* strain IK726 is a mycoparasitic fungus capable of controlling mycotoxin-producing *Fusarium* species, including *F. graminearum* and *F. culmorum*, known to produce Zearalenone (ZEA) and Deoxynivalenol (DON). DON is a type B trichothecene known to interfere with protein synthesis in eukaryotes. ZEA is a estrogenic-mimicing mycotoxin that exhibits antifungal growth. *C. rosea* produces the enzyme zearalenone hydrolase (ZHD101), which degrades ZEA. However, the molecular basis of resistance to DON in *C. rosea* is not understood. We have exploited a genome-wide transcriptomic approach to identify genes induced by DON and ZEA in order to investigate the molecular basis of mycotoxin resistance *C. rosea*.

**Results:**

We generated DON- and ZEA-induced cDNA libraries based on suppression subtractive hybridization. A total of 443 and 446 sequenced clones (corresponding to 58 and 65 genes) from the DON- and ZEA-induced library, respectively, were analysed. DON-induced transcripts represented genes encoding metabolic enzymes such as cytochrome P450, cytochrome c oxidase and stress response proteins. In contrast, transcripts encoding the ZEA-detoxifying enzyme ZHD101 and those encoding a number of ATP-Binding Cassette (ABC) transporter transcripts were highly frequent in the ZEA-induced library. Subsequent bioinformatics analysis predicted that all transcripts with similarity to ABC transporters could be ascribed to only 2 ABC transporters genes, and phylogenetic analysis of the predicted ABC transporters suggested that they belong to group G (pleiotropic drug transporters) of the fungal ABC transporter gene family. This is the first report suggesting involvement of ABC transporters in ZEA tolerance. Expression patterns of a selected set of DON- and ZEA-induced genes were validated by the use of quantitative RT-PCR after exposure to the toxins. The qRT-PCR results obtained confirm the expression patterns suggested from the EST redundancy data.

**Conclusion:**

The present study identifies a number of transcripts encoding proteins that are potentially involved in conferring resistance to DON and ZEA in the mycoparasitic fungus *C. rosea*. Whilst metabolic readjustment is potentially the key to withstanding DON, the fungus produces ZHD101 to detoxify ZEA and ABC transporters to transport ZEA or its degradation products out from the fungal cell.

## Background

The Fusarium head blight disease of cereals is caused by members of the *Fusarium* species complex, including *F. graminearum*, *F. culmorum*, *F. avenaceum* and *F. poae*[1]. These *Fusarium* spp. are well known for their ability to produce a plethora of secondary metabolites, some of which act as mycotoxins since they possess the ability to affect animals and humans adversely. Deoxynivalenol (DON) and Zearalenone (ZEA) are among the most predominant mycotoxins commonly found in infected seeds and grains [2]. DON belongs to the type B group of trichothecenes and is produced ubiquitously during plant infection where it can act as a virulence factor [3]. DON is a potent protein synthesis inhibitor which binds eukaryotic ribosomes and hampers protein translation [4,5]. DON repressed the activity of the cell wall degrading enzyme N-acetyl-beta-D-glucosaminidase in the biocontrol fungus *Trichoderma atroviride*, proposing an additional role of DON in *Fusarium* competitiveness besides being a disease virulence factor [6]. ZEA is a non-steroidal mycoestrogenic toxin that is produced largely by *F. graminearum*, *F. culmorum* and *F. equiseti*[7]. The molecular structure of ZEA resembles that of the mammalian hormone 17β-estradiol, thus consumption of the toxin by mammals stimulates hypoestrogenic responses and can result in infertility and has also been linked to cancer [4,8]. Limited information about the biological roles of ZEA is available, although it has been speculated that ZEA has functions in binding and activation of the K^+^ channel β subunit, involved in a signal transduction pathway [9]. ZEA has been shown to possess antifungal properties propounding the hypothesis that ZEA is synthesized to increase competitiveness with other fungi inhabiting the same niche [10].

Microbial detoxification of DON and ZEA has been observed by various organisms and distinct mechanisms are involved. For example, *Aspergillus* spp. disarmed the toxic effects of ZEA by conversion of the toxin to zearalenone-sulphate [11]. The yeast *Trichosporon mycotoxinivorans* was proposed to transform ZEA by cleaving a lactone ring backbone in the similar way to the detoxifying mechanism described from *Clonostachys rosea* that relies on action of the enzyme zearalenone hydrolase (EC 3.1.1.-; alternative: zearalenone lactonase, zearalenone lactonohydrolase; ZHD101) [12-14]. A recent finding reported an ability to degrade ZEA in the bacterium *Rhodococcus pyridinivorans*, although the detoxification mechanism of the strain is not yet known [15]. On the other hand, peroxidase was speculated to degrade DON in *A. oryzae* and *Rhizopus oryzae*[16] whereas hydrolytic breakdown of DON was found in *A. tubingensis*[17]. Several bacteria have also been found to be capable of detoxifying DON. These included a *Bacillus* sp., *Lactobacillus pentosus*, *L. paracasei* and *L. plantarum*[18]. Thus, de-epoxidation, epimerization and mineralization are reviewed as three important DON detoxifying mechanisms in microorganisms [19-21].

*Clonostachys rosea* (Teleomorph: *Bionectria ochroleuca*) is an ascomycete fungus with a wide range of lifestyles. The fungus has been reported to live as a saprophyte, as a nematophagous fungus and as a mycoparasitic fungus [22,23]. The *C. rosea* strain IK726 is a mycoparasitic fungus that is effective in controlling plant pathogens, including *Alternaria* spp. – the causative agent of black rot of carrot, *Bipolaris sorokiana –* the causative agent of spot blotch of barley and DON- and ZEA-producing *F. culmorum*[22-24]. Despite showing tolerance to DON, the underlying mechanism in the fungus remains obscure. In this study, we aimed to understand mechanisms regulating resistance to DON and to investigate whether additional mechanisms are involved in resistance to ZEA besides the well-known ZHD101 in *C. rosea*. We generated cDNA libraries enriched in genes expressed during interaction with DON and ZEA. Induction of genes by DON and ZEA were subsequently confirmed by the use of quantitative reverse transcription real-time PCR (qRT-PCR). Analysis of our functional annotation data suggests that metabolic readjustment is a major component for DON tolerance and ATP- Binding Cassette (ABC) transporters are involved in providing tolerance to ZEA, in addition to ZHD101.

## Results

### Construction of DON- and ZEA-induced cDNA libraries

Of 480 sequenced clones from the DON- and ZEA-induced libraries, 443 and 446 high-quality ESTs were obtained after sequence cleansing, respectively. BLASTX of the DON-induced 443 ESTs yielded 230 unigene EST sets representing 161 genes with similarity to characterized proteins (E-value ≤ 10^-6^), 82 ESTs with hypothetical proteins and 131 transcripts with either no similarity to known sequences (e-value ≥ 10^-6^). The 446 high quality ESTs from the ZEA library represented 334 unigene EST sets, BLAST searches returned 412 ESTs with similarity to characterized proteins, 4 ESTs matched hypothetical proteins and 30 ESTs showed no similarity.

**Figure 1:**
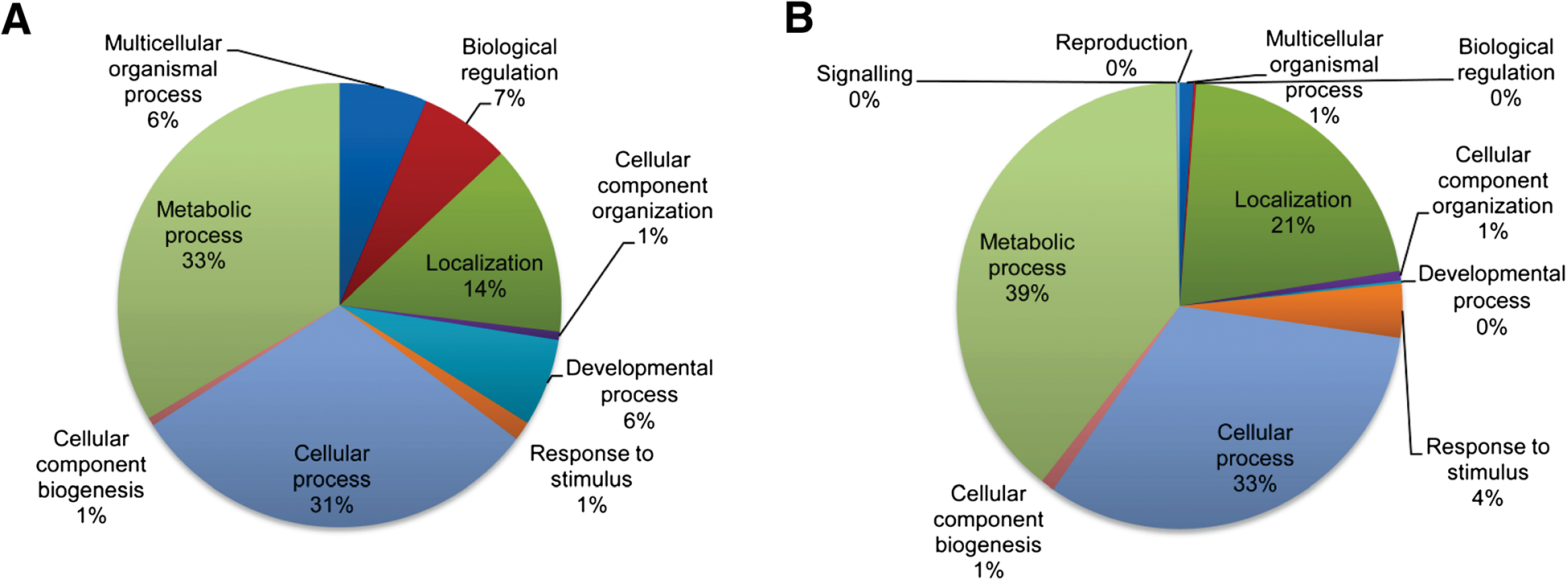
**Functional classification of Clonostachys rosea ESTs.** Distribution of putative function of ESTs with similarity to characterized proteins from **(A)** DON-induced library and **(B)** ZEA-induced library according to GO terms.

Up to 92% of the ESTs with similarity to characterized proteins from the DON-induced library were assigned a GO functional annotation. The major clusters were metabolic process and cellular process which represented 33% and 31% of the total ESTs, respectively. Similarly, analysis of 396 ZEA-induced ESTs with similarity to characterized proteins showed that metabolic process was the largest cluster accounting for 39% of the total ESTs followed by cellular process and localization at 33% and 21%, respectively. Distribution of ESTs from each library based on putative functional annotation is shown in Figure 1. The ESTs from DON- and ZEA-induced libraries were deposited at DNA Data Bank of Japan (DDBJ) under accession nos. FY998777-FY998944 and FY998945-FY999086, respectively. The ESTs that were present in high redundancy (≥ 4 times) are listed in Tables 1 and 2 for DON- and ZEA-induced libraries, respectively. Complete putative annotation and best BLAST hit are presented in Additional files 1 and 2.

**Table 1:** Transcripts that present in high frequency in DON-induced cDNA library

**Annotated transcripts**	**E-value**	**Count**	**Organism**	**DDBJ accession**
**Metabolism**				
ATP synthase alpha chain mitochondria precursor	2e^-64^	14	*Sclerotinia sclerotiorum*	FY998859
Cytochrome C oxisase polypeptide VIb	7e^-43^	12	*Trichoderma reesei*	FY998809
Acyl-CoA desaturase	2e^-136^	9	*Gibberella zeae*	FY998863
Cytochrome P450 55A3	5e^-122^	7	*Fusarium lichenicola*	FY998833
Alcohol dehydrogenase	9e^-109^	6	*Trichoderma virens*	FY998868
Glycoside hydrolase family 76 protein	3e^-43^	6	*Trichoderma virens*	FY998816
Diacylglycerol o-acyltransferase 2b	1e^-103^	5	*Trichoderma reesei*	FY998849
**Transport**				
Plasma membrane ATPase (H^+^-ATPase)	9e^-80^	22	*Trichoderma reesei*	FY998804
High affinity glucose transporter SNF3	1e^-16^	11	*Verticillium dahlia*	FY998819
Hexose transporter-like protein (TrHXT2)	2e^-141^	6	*Trichoderma reesei*	FY998842
**Stress response**				
Major allergen asp f2-like protein	7e^-23^	6	*Metarhizium anisopliae*	FY998813
Mitochondrial hypoxia responsive protein	7e^-45^	6	*Trichoderma atroviride*	FY998834
**Cell cycle**				
ThiJ/PfpI family protein	1e^-131^	29	*Metarhizium acridum*	FY998826
CHK1 checkpoint-like protein	2e^-16^	9	*Trametes versicolor*	FY998823
Eukaryotic initiation factor 1 SUI1	4e^-59^	8	*Gibberella zeae*	FY998855
Glucose repressible protein grg1	7e^-29^	5	*Beauveria bassiana*	FY998852

**Table 2:** Transcripts that present in high frequency in ZEA-induced cDNA library

**Annotated transcripts**	**E-value**	**Count**	**Organism**	**DDBJ accession**
**Metabolism**				
Zearalenone hydrolase	1e^-176^	120	*Bionectria ochroleuca*	FY998945
Glycoside hydrolase family 5	4e^-125^	21	*Trichoderma virens*	FY998957
Amidophosphoribosyltransferase	9e^-65^	15	*Magnaporthe oryzae*	FY998962
Cytochrome P450	1e^-62^	8	*Trichophyton verrucosum*	FY998953
Dihydrolipoyl dehydrogenase	3e^-32^	5	*Metarhizium anisopliae*	FY998966
4-aminobutyrate aminotransferase	3e^-16^	5	*Verticillium dahlia*	FY998971
Pyruvate decarboxylase	9e^-50^	4	*Trichoderma reesei*	FY998999
**Transport**				
ABC transporter CDR4	5e^-74^	21	*Neurospora crassa*	FY999079
multidrug resistance protein CDR1	4e^-93^	19	*Colletotrichum higginsianum*	FY999081
ABC transporter	4e^-33^	18	*Gibberella pulicaris*	FY999076
ABC transporter	8e^-104^	13	*Nectria haematococca*	FY999078
multidrug resistance protein CDR1	2e^-52^	8	*Colletotrichum higginsianum*	FY999084
ABC transporter	8e^-33^	8	*Gibberella pulicaris*	FY999082
ABC transporter	1e^-113^	6	*Nectria haematococca*	FY999077
pleiotropic drug resistance protein TABC2	7e^-32^	6	*Trichoderma atroviride*	FY999083
Bacteriorhodopsin	6e^-14^	6	*Colletotrichum higginsianum*	FY998978
Major facilitator superfamily transporter	8e^-39^	5	*Glomerella graminicola*	FY998969
Vascuolar protein sorting 26	9e^-49^	5	*Metarhizium anisopliae*	FY999008
Allantoate permease of major facilitator superfamily	2e^-38^	5	*Glomerella graminicola*	FY999011
Mitochondrial phosphate carrier protein	2e^-28^	4	*Trichoderma atroviride*	FY999030
Protein CCC1	1e^-16^	4	*Metarhizium anisopliae*	FY999002
Pleiotropic Drug Resistance family protein	1e^-72^	4	*Trichoderma reesei*	FY999085
**Stress response**				
Heat shock protein 70	2e^-39^	8	*Nicotiana tabacum*	FY998983
**Cell cycle**				
FACT complex subunit pop3	4e^-47^	7	*Nectria haematococca*	FY998996
GTP binding protein ychF	7e^-142^	5	*Metarhizium acridum*	FY998981
Prohibitin phb1	6e^-147^	4	*Metarhizium anisopliae*	FY998992

### Highly redundant *C. rosea* genes in the DON-induced library

A set of transcripts induced by DON were classified with putative functions in metabolism, cell cycle, transport and stress response. The majority of the redundant transcripts putatively encoded metabolic or biosynthetic enzymes, for instance, 7 of cytochrome P450 55A3 (CYP450 55A3; EC:1.14.-.-), 12 of cytochrome C oxidase subunit Vib (COX; EC:1.9.3.1), 5 of diacylglycerol o-acyltransferase (EC:2.3.1.20), 9 of acyl-CoA desaturase (EC:1.14.19.1), and 6 of glycoside hydrolase family 76 (GH76; EC:3.2.1.-).

Other redundant transcripts putatively encoded proteins involved in the cell cycle. ThiJ/PfpI protein family was among the most highly induced ESTs in the DON-induced library, being found 29 times. In addition, ESTs encoding high affinity glucose transporter SNF3, hexose transporter-like protein and plasma membrane ATPase (H^+^-ATPase; EC:3.6.3.6) exhibited increased in expression. We also observed high redundancy for ESTs encoding proteins associated with stress responses. These included molecular chaperones heat shock protein HSP70 and HSP90, mitochondria hypoxia responsive domain protein and flavohemoglobin.

### Highly redundant *C. rosea* genes in the ZEA-induced library

Analysis of the ZEA-induced library revealed that the majority of transcripts with high redundancy encoded ZHD101 and ABC transporters resembling Candida Drug Resistance (CDR)1 and CDR4 of *Candida albicans* and ABC-2 type transporters. In addition to ZHD101, ESTs putatively encoding other metabolic enzymes were recorded including CYP450 and amidophosphoribosyltransferase (EC:2.4.2.14). ESTs putatively encoding enzymes involved in glycolysis and TCA such as pyruvate kinase (EC:2.7.1.40), aconitrate hydratase (EC:4.2.1.3) and pyruvate decarboxylase (EC:4.1.1.1) were also present in high numbers in the ZEA-library.

In addition, we found ESTs encoding glycoside hydrolase family 5 (GH5) that exhibits broad known activities, including glucan β-1,3-glucosidase (EC: 3.2.1.58), β-mannosidase (EC: 3.2.1.25) and chitosanase (EC:3.2.1.132), and other ESTs encoding proteins regulating the cell cycle, *e.g.*, Facilitating Chromatin Transcription (FACT) complex subunit pob3, GTP binding protein (GTPase) *ychF* and prohibitin presented in high redundancy in the ZEA-induced library. We also noted transcripts encoding Major Facilitator Superfamily (MFS) transporter induced by ZEA.

### Phylogenetic analysis of ABC transporters detected in the ZEA-induced library

**Figure 2:**
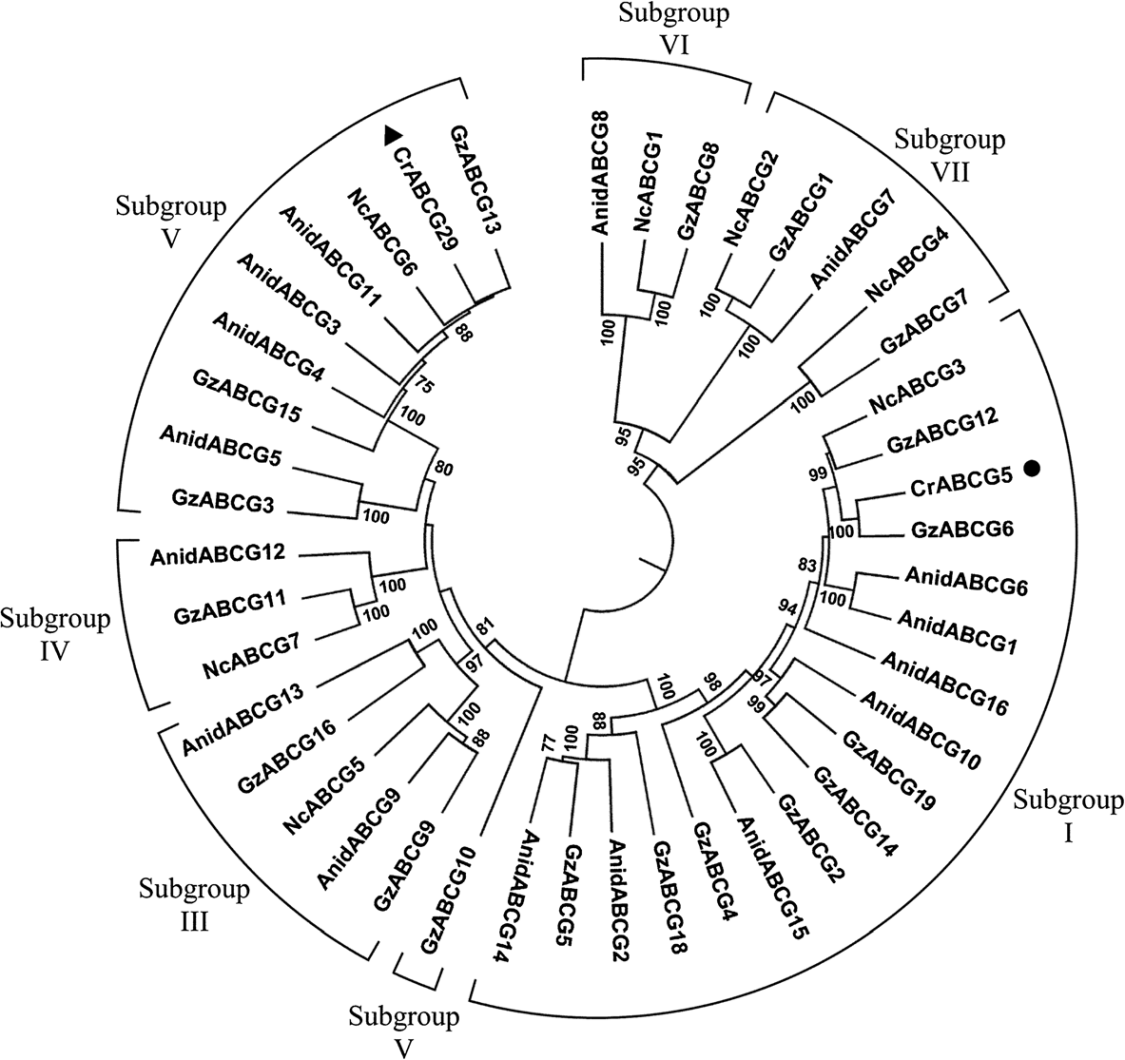
**Phylogenetic analysis of fungal subgroup G ABC-transporters.** The displayed tree showed only the clade where the two predicted genes – ABCG29 (closed triangle) and ABCG5 (closed circle) – were clustered. ABC-G subgroups were designated according to [25]. Other ABC transporters which were included to generate the tree were from Aspergillus nidulans (Anid), Gibberella zeae (Gz) and Neurospora crassa (Nc). Bootstrap support values (≥ 70%) are associated with branches.

Local BLAST searches to the draft *C. rosea* IK726 genome sequence revealed that all ESTs from the ZEA-induced library exhibited similarity to only two different ABC-transporter genes. The bioinformatic tool FGENESH + was further employed to predict two full-length ABC transporters with 1436 and 1321 amino acids, respectively. Full-length nucleotide sequences of the two predicted genes were shown in Additional file 3. We performed phylogenetic analysis to investigate whether the identified ABC transporters pertain to xenobiotic-transport classes of ABC proteins. The analysis revealed that they belong to group G of fungal ABC transporters (Figure 2) [25] and the two genes were named *abcG5* and *abcG29* according to the nomenclature for fungal ABC transporters [25]. Group G consists of 7 different subgroups in which most of them harbour relevant functions to either xenobiotic or drug transport. Our analysis indicated that the ABCG5 belong to subgroup I which is related to multidrug resistance, whereas ABCG29 belong to subgroup V which contains members of unknown function.

### Gene expression of selected genes from the DON- and ZEA-induced libraries

**Figure 3:**
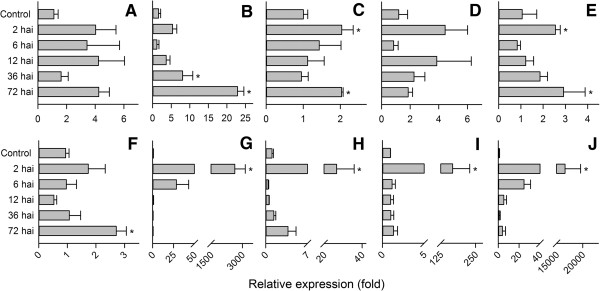
**Gene expression of Clonostachys rosea genes.** Validation and temporal expression of the selected genes from DON- **(A-E)** and ZEA-induced **(F-J)** cDNA library. The selected genes for DON-induced library were encoding hexose transporter-like protein **(A)**, CYP450 **(B)**, diacylglycerol o-acyltransferease **(C)**, ThiJ/PfpI family protein **(D)**, pyruvate decarboxylase **(E)**, and major facilitator protein **(F)**, zhd101 **(G)**, GH5 **(H)**, abcG5 **(I)** and abcG29 **(J)** for ZEA-induced library. The asterisks indicated significant difference (P ≤ 0.05) of expression in comparison to control treatment using Turkey’s multiple range tests following ANOVA analysis.

To validate genes induced by DON and ZEA, we performed qRT-PCR on 5 selected genes from each library at 2, 6, 12, 36 and 72 hours after inoculation. This temporal gene expression set-up would allow us to monitor the expression dynamics of the candidate genes. In this study, we chose expression of the candidate genes at 2 hours as the calibration point as we foresaw an immediate response of the fungus to ZEA. Analysis with qRT-PCR showed that all the selected genes exhibited a rapid response to both DON and ZEA (Figure 3). After 2-hour exposure to ZEA, the expression of transcripts encoding ZHD101 and ABCG29, identified in the ZEA-induced library, accumulated sharply to more than a thousand fold (*p* ≤ 0.05). Another ABC transporter encoded by *abcG5* was induced 186-fold (*p* ≤ 0.05). On the other hand, the selected transcripts from the DON-induced library were induced to a lesser extent. Up to 3- and 2-fold change in expression were detected for pycruvate decarboxylase (*p* ≤ 0.05) and diacylglycerol o-acyltransferease after 2-hour exposure to DON, respectively. Temporal gene expression revealed a substantial drop in expression of most selected genes from ZEA-induced library after 6 hours of exposure whereas 3 of 5 selected genes from DON-induced library were increased in expression at 72 hours (Figure 3). The DON-induced genes with significant alteration in expression (*p* ≤ 0.05) at 72 hours included CYP450 (22 fold), diacylglycerol o-acyltransferease (2 fold) and pyruvate decarboxylase (2.8 fold).

## Discussion

Tolerance to xenobiotics is of importance for antagonistic fungi during interactions with plant pathogens that produce a broad range of secondary metabolites. Our aim with the current study is to advance our understanding of mechanisms conferring resistance to *Fusarium* mycotoxins DON and ZEA in the hyperparasitic fungus *C. rosea* IK726 that is effective in controlling *Fusarium* species. For this, we employed a genome-wide transcriptomic approach based on suppression subtractive hybridization to explore molecular responses of the fungus towards DON and to ZEA. The analysis of the DON-induced transcripts does not suggest additional mechanisms compared to those previously discovered to render tolerance to DON in microorganisms [19]. This suggests to us that resistance to DON in *C. rosea* is complex and is the result of synergistic action of proteins from different pathways rather than a stand-alone mechanism. The analysis of ZEA-induced transcripts suggest that thatZHD101, previously reported as a key enzyme regulating resistance to ZEA in *C. rosea*, and 2 ABC transporters may be involved in ZEA resistance.

Metabolic readjustment may be a major component in DON tolerance in *C. rosea*, as transcripts encoding metabolic enzymes such as CYP450 55A3, COX and mitochondrial ATP synthase are identified in high frequency. Involvement of these enzymes in abiotic stress tolerance has been reported previously. For example, overexpression of COX improved resistance to the antimicrobial peptide MiAMP1 in *Saccharomyces cerevisiae*[26]. A membrane associated ATP synthase is highly induced in a Cercosporin-resistant *Cercospora nicotianae* strain but not in a susceptible strain [27].

Exposure to DON induced expression of transcripts encoding a number of transporters. These included the high affinity glucose transporter SNF3, the hexose transporter-like protein TrHXT2 and a plasma membrane H^+^-ATPase. In *S. cerevisiae*, SNF3 is a glucose sensor that generates a intracellular glucose signalling cascade required for induction of hexose transporter expression, whereas HXT1 (orthologous to TrHXT2) is a high-affinity glucose and mannose transporter [28]. The presence of ESTs encoding proteins similar to SNF3 and TrHXT2 suggests that the demand of cellular energy is increased during DON exposure. Taken together with up-regulation of genes encoding metabolic enzymes as mentioned above, it is possible that the increased need of cellular energy is to produce proteins to compensate those destroyed by DON. This idea is supported by the up-regulation of a gene that putatively encodes a proton transporter H^+^-ATPase, which is shown to facilitate the uptake of nutrients by providing proton gradients for membrane transporters, and to regulate intracellular pH [29-31]. Interestingly, we also observed the accumulation of transcripts putatively encoding enzymes in the triglyceride synthesis pathway. Triglycerides may act as an energy reservoir and the specific induction of by DON, but not by ZEA, provide further support for an increased energy demand during DON exposure.

DON has been shown to generate a substantial level of reactive oxygen species (ROS) and oxidative stress, which can induce protein damage and DNA strand breakage in human HepG2 cells [32]. This might explain the up-regulation of genes encoding stress-response proteins such as the chaperones (heat shock proteins, Hsp) 70 and Hsp90 subunit that possess several important cytoprotective functions, including prevention of protein aggregation and degradation of unstable proteins [33], and the cell cycle checkpoint protein (Chk1) that is essential for cellular function in response to DNA damage [34,35]. Hsp70 and Hsp90 transcripts often accumulate following exposure to biotic and abiotic stresses in several organisms [33,36,37]. As DON generates oxidative stress that damages proteins and DNA, it is likely that Chk1is triggered to protect *C. rosea* from DNA damage by the toxin, while the Hsp70/90 subunits act to protect or recycle damaged proteins.

A previous study showed that alterations in cell wall structure are connected with increased resistance to a killer toxin in *S. cerevisiae*[38]. DON-exposure induce genes that putatively encode a GH76 and a GH22 enzyme, with known α-1,6-mannanase (EC 3.2.1.101) and dolichol-P-mannose α-mannosyltransferase (EC 2.4.1.-) activity, respectively. These activities are reported to be involved in incorporation of glycoproteins into the cell wall of *N. crassa* and S. *cerevisiae*[39,40]. ZEA-exposure on the other hand induces a putative endoglucanase (EC 3.2.1.-) GH5 gene that is involved in cell wall modification in *A. nidulans*[41]. We may therefore hypothesise that cell wall modifications are part of the resistance machinery to both DON and ZEA toxins in *C. rosea*.

ZEA tolerance in *C. rosea* was determined by ZHD101 that cleaves off one of the lactone rings in the backbone, resulting in the product 1-(3,5-dihydroxyphenyl)-10′-hydroxy-1-undecen-6′-one, and that the structural change resulted in reduced toxicity of ZEA [12]. In our study, qRT-PCR analysis showed that the fungus responded to ZEA as early as 2 hai and expression of *zhd101* decreased significantly at least 200 fold when measured at later time points. This confirms that the time point chosen for library construction was accurate and also supports the previous finding of ZHD101 as a player in ZEA tolerance. Surprisingly, we noticed an increase in expression of transcripts encoding the molecular chaperone Hsp70 in the ZEA-induced library. The fact that the fungus was stressed during exposure to ZEA, despite possessing ZHD101, suggests that ZHD101 is not the only mechanism underlying resistance to ZEA in *C. rosea*.

ABC transporters are major secondary transport systems that render resistance to xenobiotics in organisms [25,42]. We hypothesise from the abundant ESTs encoding ABC transporters in the ZEA-induced library that ABC transporters – together with ZHD101 – contribute to resistance to ZEA in *C. rosea* through the significant increase in transcript levels for ABC transporters from group G (ABC-G) of fungal ABC transporters, which are well known for contributing to drug/fungicide resistance in many fungi [25]. This is supported by the concomitant expression of the two ABC-G proteins have with ZHD101. This is the first report to suggest that the ABC transporters are potentially involved in providing resistance to ZEA. Previously, Kakeya and colleagues demonstrated that the product of ZHD101 activity on ZEA, namely 1-(3,5-dihydroxyphenyl)-10′-hydroxy-1-undecen-6′-one, did not possess any estrogenic potencies to human breast cancer MCF-7 cells [12]. Nonetheless, it is unclear whether this degradation product 1-(3,5-dihydroxyphenyl)-6′-hydroxy-1-undecen-10′-one possesses toxic activities and thus it triggers expression of the ABC transporters or whether the ABC proteins act as ZEA efflux pump preventing cells from being damaged from ZEA when ZHD101 is degrading the toxin.

Group G of fungal ABC transporters comprises 5 subfamilies [25], and our phylogenetic analysis of the predicted full-length *C. rosea* ABC transporters suggested that the transporters belong to the subgroup I (ABCG5) and subgroup V (ABCG29) of subfamily G. The subgroup I is well known for contributing resistance to drugs and fungicides in fungi, and includes Pdr5p and Pdr10p from *S. cerevisiae* and Cdr1p, Cdr2p, Cdr3p and Cdr4p proteins from *C. albicans*. While functions of the subgroup I of ABC-G proteins has been investigated thoroughly, information about the subgroup V of fungal ABC-G is limited, including their biological roles. The similar expression patterns of the two ABC transporters with that of *zhd101* suggests that these *C. rosea* ABC transporters evolved as a specific mechanism to withstand ZEA, potentially by providing efflux of ZEA and/or its digested products.

## Conclusions

In conclusion, our SSH results suggested that tolerance to DON in *C. rosea* is provided by a consort of enzymes and proteins, covering a broad range of genes from metabolism to transporters. Cellular energy is manipulated to generate proteins to compensate for those that are destroyed by DON. This is ascertained by the increase in transcripts encoding (I) metabolic-related enzymes such as CYP450 and COX, (II) sugar transports such as HXT2 and H^+^-ATPase and (III) cellular response such as Hsp70 and Hsp90. On the other hand, two ABC transporters may participate in conferring resistance to ZEA together with ZHD101. This is the first time that participation of ABC transporters in ZEA detoxification are implicated, which was thought previously to rely only on ZHD101.

## Methods

### Fungal cultures

*C. rosea* strain IK726 was revived from −80°C glycerol stock on Czapek-Dox agar (Merck) for 5 days at room temperature. A plug of actively growing mycelium was subsequently transferred to 25 ml Czepak-Dox broth (Sigma) in 250 ml Erlenmeyer flask and incubated at 25°C for 5 days prior to toxin treatment.

Pure DON (cat. no. D0156) and ZEA (cat. no. Z2125) were purchased from Sigma-Aldrich and dissolved in methanol before storing at −20°C as a stock. DON- or ZEA-containing methanol was applied separately into the culture medium to achieve a final concentration of 5 and 10 ppm, respectively. An equal amount of methanol was incorporated in the control experiment. After the treatments, the cultures were incubated for 72 and 2 hours for DON and ZEA treatment, respectively, at 25°C on a 150 rpm rotary shaker before harvesting mycelium by vacuum filtration. The harvested mycelium was flash frozen with liquid nitrogen and stored at −80°C until use.

### RNA extraction and construction of DON- and ZEA-induced subtractive cDNA libraries

Total RNA was extracted from DON-, ZEA-treated and control samples using Spectrum™ Plant total RNA kit (Sigma) according to the manufacturer’s protocol. To ensure the absence of DNA impurities, removal of residual DNA was achieved by on-column DNA digestion RNase-Free DNase Set (Qiagen) following the manufacturer’s protocol. The RNA obtained was quantified and monitored for quality by Nanodrop spectrophotometer ND-1000 (Thermo Scientific). Subsequently, mRNA was extracted from 100 μg total RNA by Dynabeads® mRNA Purification Kit (Invitrogen) before proceeding with subtractive hybridization.

750 ng mRNA from DON-, ZEA-treated and control samples was used to generate each subtractive cDNA library. Synthesis of double-stranded cDNA for all treatments and suppression subtractive hybridization (SSH) utilised PCR select™ Subtractive Hybridization Kit (Clontech) according to the manufacturer’s protocol. Only forward subtraction was performed with DON- or ZEA-treated mRNA as the driver and control treatment mRNA as the tester for each library.

Amplification of the subtracted transcripts was performed using Advantage Taq polymerase (Clontech). A 2 μl aliquot of the PCR product obtained from each library were cloned into the pCR®II-TOPO® vector using TOPO TA cloning kit (Invitrogen) before subsequent transformation to Library Efficiency® DH5α™ chemical competent cells (Invitrogen). Colony PCR of a total of 480 randomly picked clones from DON- and ZEA-subtracted cDNA libraries was performed using M13 primers and Hotmaster® Taq DNA Polymerase (5 PRIME) on Gene Amp PCR system 2400 (Applied BioSystem). The PCR products were purified using QIAquick® PCR purification kit (Qiagen) according to the manufacturer’s protocol and were subject to gel electrophoresis with 1% agarose. PCR products larger than 200 base pairs were collected and sequenced using Applied Biosystems 3730XL Sanger sequencing with BigDye terminator serviced by Beckman Coulter genomics (Essex, United Kingdom).

### Sequence analysis and annotation

A total of 480 sequences acquired from each library were cleansed and trimmed to remove a vector backbone and assembled using the software package CLC Main Workbench version 6.5 (CLC Bio, Denmark). BLASTX [43] was adopted to search for similar non-redundant proteins in GenBank protein database [44] using the BLAST function of CLC Main Workbench with the cut-off E-value of 10^-6^. Sequences with no significant hit from BLASTX were subjected to BLASTN against nr/nt nucleotide collection of the GenBank with the cut-off E-value of 10^-6^. Functional annotation of ESTs with significant database matches was performed using BLAST2GO where the Gene Ontology (GO) annotation of level 2 biological process was achieved [45]. The GO annotation was analysed using default settings with and E-value threshold = 10^-6^.

### Quantitative reverse transcription – polymerase chain reaction (qRT-PCR) analysis for validation of SSH results

To validate genes up-regulated during exposure to DON or ZEN, qRT-PCR was performed with five genes from each library selected for their putative involvement in secondary metabolite resistance or because of their high level of EST redundancy. Fungal culture and inoculation of the toxins were carried out in triplicate as a separate experiment as mentioned above except that fungal mycelia were collected temporally at 2, 6, 12, 36 and 72 hours after inoculation (hai). The mycelia were immediately flash frozen with liquid nitrogen and were kept at −80°C until use.

Total RNA extraction was conducted using RNeasy Plant Mini Kit (Qiagen) prior to RiboLock RNase inhibitor (Fermentas) and DNase I (Fermentas) treatment to remove DNA contaminants following the manufacture’s protocol. The total RNA was quantified with Nanodrop spectrophotometer (Thermo Scientific). First-strand cDNA was synthesised from 1 μg total RNA using iScript cDNA synthesis kit (BIO-RAD) with random primers according to the manufacturer’s protocol. Gene expression analysis was performed in two technical replicates for each biological replicate on a Mx3000P qPCR system (Stratagene) with 150 ng of cDNA and Maxima® SYBR Green qPCR Master Mix (Fermentas). A selected set of gene-specific primers for DON- and ZEA-induced cDNA libraries was listed in Table 3. Analysis of melting curves was carried out at the end of each run to evaluate undesired amplifications. Expression of *tub2* gene encoding β-tubulin that was previously evaluated to be constitutively and constantly expressed in *C. rosea*[46] was used as a housekeeping gene to normalise target gene data. Subsequently, temporal expression of each selected genes at all time points were compared to its expression at 2 hours using the 2^-ΔΔ^CT relative gene expression method [47]. This was done to achieve an overview of gene expression dynamics in comparison to the earliest response at 2 hours. Gene expression data were analysed statistically using analysis of variance (ANOVA) with a General Linear Model implemented in MINITAB version 15 (Minitab Inc.). Pairwise comparisons were made using Tukey’s method with a confidential level of 95%.

**Table 3:** List of primers used in the qRT-PCR validation

**Target gene**	**Forward primer**	**Reverse primer**
**DON-induced library**		
β-tubulin	GGTCAGTGCGGTAACCAAAT	ACAGCGCGAGGAACATACTT
Hexose transporter-like protein	GCGCAACATCCGGCAAAAA	CTCGCTTGGGCTGTGAAT
CYP50 NOR	CTTGTGGTTGAGCAGCTT	ATGTTCTGGGTGTTGCAT
ThiJ/PfpI family protein	ATTCTCATCCTCGTCACCC	ACAACCCACCCCGATTATA
Diacylglycerol o-acyltransferase	AGCGTCAATAAGGTGTTGG	GAAGCTACACAGGACGCA
Pyruvate decarboxylase	CCCAACCAAGTCCATCTGT	GTGTCCCAGATGCCAAAGT
**ZEA-induced library**		
β-tubulin	GGTCAGTGCGGTAACCAAAT	ACAGCGCGAGGAACATACTT
Zearalenone hydrolase	GTGCCACGAACTGCCAACAAAG	CGCCTCCGAGCCTCCAGACAC
abcG29	CAGCCCCGAGTTTAGCAA	GGTATTTTGCTCTGCCTCTG
abcG5	GTCAACTTGGGCTTCGAATG	CCTCACTGTTCTTCCAGC
Major facilitator transporter	ATCCCATTACCAACGCCA	ACTCCGAGGAAGAATCGC
Glycoside hydrolase family 5	CGCACGTGAACAATCCTC	ACGAAGATCAGCCAGTCC

### Bioinformatics prediction of the ZEA-induced full-length ABC transporters

Full-length sequences of ZEA-induced ABC transporters were predicted from an Illumina- and SOLiD-based draft genome assembly of the *C. rosea* IK726 genome (Karlsson *et al*., unpublished) using FGENESH + [48] and a *F. graminearum* ABC transporter (locus FGSG_03735) as the template. To categorize the ABC transporters, amino acid sequences of known fungal ABC transporters from *F. graminearum*, *A. nidulans* and *Neurospora crassa*[25] were used as references to generate a phylogenetic tree. Sequences were aligned by ClustalW [49] implemented in the Molecular Evolution and Genetics Analysis (MEGA) software package version 5 [50]. Phylogenetic analyses were performed using Neighbour-Joining implemented in MEGA5, using pairwise deletion of gaps and the Poisson correction distance of substitution rates. Statistical support for phylogenetic grouping was estimated by 1000 bootstrap resamplings.

## Availability of supporting data

The data sets supporting the results of this article are included within the article and its additional files.

## Competing interests

The authors declare that they have no competing interests.

## Authors’ contributions

CK performed the experiments, bioinformatics and transcriptome analyses, participated in the design of the study and drafted the manuscript. MK performed bioinformatics analysis, participated in the design of the study and the draft manuscript. AD participated in transcriptome analysis and drafted the manuscript. DFJ and DBC conceived the study, participated in its design and coordination. All authors have contributed to writing the manuscript. All authors read and approved the final manuscript.

## Supplementary Material

Additional file 1 DON-induced library. Click here for file

Additional file 2 ZEA-induced library. Click here for file

Additional file 3 Predicted ABC transporters. Click here for file
